# Interleukin-35 mitigates the function of murine transplanted islet cells via regulation of Treg/Th17 ratio

**DOI:** 10.1371/journal.pone.0189617

**Published:** 2017-12-13

**Authors:** Yin Zongyi, Zou Funian, Li Hao, Wang Xin, Cheng Ying, Zhang Jialin, Liu Yongfeng, Li Baifeng

**Affiliations:** 1 Department of Hepatobiliary Surgery and Organ Transplantation, the First Hospital of China Medical University, Shenyang, China; 2 Department of Hepatobiliary Surgery, Shenzhen University General Hospital, Shenzhen, China; 3 National Key Lab. of General Surgery, the First Hospital of China Medical University, Shenyang, China; 4 Multiple Organ Transplantation Institute of the First Hospital of China Medical University, Shenyang, China; Children's Hospital Boston, UNITED STATES

## Abstract

Pancreatic islet transplantation is a promising treatment for type 1 diabetes (T1D). Interleukin-35 (IL-35) is a recently discovered cytokine that exhibits potent immunosuppressive functions. However, the role of IL-35 in islet transplant rejection remains to be elucidated. In this study, we isolated islet cells of BALB/c mouse and purified CD4+ T cell subsets of a C57BL/6 mouse. The model for islet transplantation was established *in vitro* by co-culture of the islet cells and CD4+ T cells. IL-35 (20 ng/ml) was administered every other day. Following co-culture, the islet function and Treg/Th17 ratio were analyzed on days 1, 3, and 5. Furthermore, the Th17/Treg ratio was modulated (1:0–2), and the function of islet cells as well as proliferation of Th17 cells were analyzed. T cell sorting was performed using the magnetic bead sorting method; Treg and Th17 count using flow cytometry; cell proliferation detection using the carboxyfluorescein diacetate succinimidyl ester (CFSE) method, and islet function test using the sugar stimulation test. Results showed that Th17 counts increased in the co-culture system. However, after administration of IL-35, the number of Treg cells increased significantly compared to that in the control group (50.7% of total CD4+ T cells on day 5 in IL-35 group vs. 9.5% in control group) whereas the proliferation rate of Th17 cells was significantly inhibited (0.3% in IL-35 group vs. 7.2% in control group on day 5). Reducing the Th17/Treg ratio significantly improved the function of transplanted islets. Treg inhibited Th17 proliferation and IL-35 enhanced this inhibitory effect. IL-35 mitigates the function of murine transplanted islet cells via regulation of the Treg/Th17 ratio. This might serve as a potential therapeutic strategy for in-vivo islet transplant rejection and T1D.

## Introduction

Pancreatic islet transplantation (PIT), a treatment for type 1 diabetes (T1D), is a minimally invasive procedure that can restore normoglycemia and insulin independence without surgical complications [1, 2]. Current immunosuppression strategies poses several risks (such as infection and cancer) to transplant recipients [3–7]. Although a recent report showed that in most experienced institutions, the 5-year survival rate of transplanted islets reached up to 50% [8], overall long-term results remain unsatisfactory [9].

Emerging evidences suggest that T helper 17 (Th17) and regulatory CD4+CD25+Foxp3+ T (Treg) cells have a distinct differentiation pathway, which are different from that of T helper 2 (Th2) cells or T helper 1 (Th1) cells [10–12]. Tregs play an anti-inflammatory role mainly by releasing inhibitory cytokines such as TGF-β and IL-10 or contact-dependent suppression on other immune cells, including CD8+, CD4+ T cells and B cells [12]. Increase in Tregs have been reported to be involved in the development of immune tolerance [13] and solid organ transplantation (e.g. kidney transplant [14–17], liver transplant [18–22] and heart transplant [23, 24]). In contrast, Th17 cells, mainly expressed by factors such as retinoic acid receptor-related orphan receptor γt (RORγt), have been reported to play a potent pro-inflammatory role by producing the signature cytokine IL-17A [25–29]. A series of studies have reported that Th17 cells widely contribute to autoimmune diseases and transplant rejection [26, 27, 30–34]. Recent studies found that the balance between Tregs and Th17 plays an important role in the above diseases, by regulation of the immunologic homeostasis through the secretion of anti- or pro-inflammatory cytokines, depending on the activation of Forkhead box P3 (FoxP3) and signal transducer and activator of transcription 5 (STAT5) or RORγt and STAT3, respectively [30, 31, 33, 35, 36].

IL-35, consisting of IL-12α subunits and Epstein-Barr-virus-induced gene 3 (Ebi3), is a recently discovered cytokine exhibiting potent immunosuppressive functions [37–40]. It is secreted by and contributes to the proliferation of Tregs. It not only promotes differentiation of conventional CD4+T cells into Tregs but also converts Tregs into induced regulatory T cells (iTr35); the latter lack FoxP3 expression, release IL-35 but not IL-10 or TGFβ, and possess stronger immunosuppressive properties than Tregs [35, 37–39, 41–45]. Numerous studies have concentrated on the functions of IL-35 in autoimmune and inflammatory diseases, such as psoriasis [30], T1D [41], arthritis [42], asthma [44, 46] and leukemia [47].

However, the role of the balance of Treg/Th17 and the therapeutic potential and effects of IL-35 in islet transplantation has been unclear so far. Hence, here, we aimed to clarify and examine the role of Treg/Th17 and the kinetic effects of IL-35 in an *in vitro* mouse islet transplantation model.

## Materials and methods

### Animals

All animal experiments were approved by the local animal ethics committee at the First Hospital of China Medical University. Male BALB/c and C57BL/6 mice aged 8–12 weeks and weighing 23–28 g were used for the study. The mice were supplied by the laboratory animal center of China Medical University (Shenyang, China) and raised carefully in accordance with international guidelines (National Institutes of Health 85–23) as well as the current version of the China Law on the Protection of Animals. The mice were raised in pathogen-free cages and kept at a relative humidity of 50–70% and temperature of 20–25°C. Mice was sacrificed using exsanguination method under anesthesia (1.5% sevoflurane).

### Isolation and purification of islets

Pancreatic islets were prepared by the collagenase P (Roche Diagnostics Scandinavia, Bromma, Sweden) method from overnight fasted BALB/c mice. In brief, a mouse was anesthetized with 1.5% sevoflurane and fixed in the supine position. The skin was disinfected with 75% ethanol followed by sterile laparotomy. The common bile duct (CBD) close to the duodenum was ligated for the retrograde puncture of CBD, followed by a slow perfusion of 3 mL collagenase-P (pre-chilled at 4°C) to fully expand the pancreatic body and tail. The heart was excised to drain the blood and the pancreas was recovered by blunt isolation. The isolating solution (composed of 500 mL Hanks solution containing 10 mM of HBSS and 15 mM of CaCl_2_) was filter-sterilized through a 0.22-μm filter and adjusted to pH 7.2–7.4 prior to storage at 4°C until use. The digestive solution, with a final concentration of 1 mg/mL collagenase-P, was freshly prepared before using the afore-mentioned isolating solution (pH 7.2–7.4). Ficoll-400 density gradient centrifugation was employed to purify the islets as per a previous study [48].

### Lymphocyte isolation and CD4+CD25-/+, IL-17A+ T cell sorting

Single splenic lymphocytes were isolated from C57BL/6 mice as previously described [39, 42, 49]. CD4+, CD4+CD25+, IL-17A+, and CD4+CD25- T cells were sorted by using the Stemcell magnetic sorter (cat: 18000), the human/mouse CD4 T cell negative selection kit (cat: 19852), the human/mouse Treg positive selection kit (cat: 18782), following the manufacturer’s instructions. All these instruments and kits were from Stemcell Technologies Inc, Shanghai, China.

### Flow cytometry staining

Flow cytometry analysis of CD4+, CD4+CD25+, CD4+IL-17A+ and CD4+CD25+FoxP3+ T cells were performed according to the Intracellular Cytokine Staining protocol or the Cell Surface Immunofluorescence Staining Protocol described in the T Cell Staining Kit (Biolegend, San Diego, CA, USA). All flow antibodies and relative reagents were from Biolegend: FITC-anti-human/mouse CD4 (cat: 100406), PE/Cy5-anti-human/mouse CD25 (cat: 102010), PE-anti-mouse/rat/human FoxP3 (cat: 320008), and APC-anti-human/mouse IL-17A (cat: 506916). For intracellular staining, single T cells were stimulated for 6 hours with Cell Activation Cocktail (with Brefeldin A) (2 μl/ml; cat: 423304, Biolegend, San Diego, USA). The stained cells were counted using the BD FACSCanto II. The data obtained were analyzed using Flow Jo 7.6 software (Tree Star, Inc., Oregon, USA). Gating strategies were performed following the manufacturer’s instructions (BD).

### Cell proliferation assays

We detected the proliferation of Th17, CD4+CD25- T cells (effector cells) using CFSE labeling as previously described [50]. Freshly purified T cells were re-suspended in phosphate buffer saline (0.1% BSA) at 2 x 10^6^ cells/ml and incubated with CFSE (1 μl/ml; Abcam, Cambridge, UK) for 15 min at 37°C. These cells were then washed and re-suspended in 1640 Medium for 10 min to stabilize the CFSE staining. Cells were re-suspended in the culture medium after a final wash step.

### Viability and functional assays of islet cell clumps

The glucose-stimulated insulin secretion (GSIS) assay was employed to detect the function of co-cultured islets as previously described [51]. Krebs–Ringer bicarbonate was used as the base media. The basal glucose level used was 2.5 mM, following which a glucose level of 16.7 mM was used to stimulate the islet cells. The insulin concentration of supernatant was analyzed using an enzyme-linked immunosorbent assay (ELISA) kit (Alpha Diagnostic Intl. Inc., USA) following the product manual. The insulin staining was performed as previously described [52].

### *In vitro* IL-35 and Treg treatment

For *in vitro* experiments, the cells were categorized into three groups: IL-35 group, Treg group, and control group. In the IL-35 group, approximately 50 islets and 2 x 10^5^ CD4+ T cells were co-cultured in 24-well plates. IL-35 (20 ng/ml) (cat: RPC008Mu, CLOUD-CLONE CORP., USA) was added to the plate and replenished every time the culture medium was changed (every other day). In the Treg group, we regulated the ratio of Treg and Th17 cells as 0:1, 0.5:1, 1:1, and 2:1 by increasing the amount of Tregs and co-cultured these T cells (totally 2 x 10^5^ cells) and 50 islets in 24-well plates, separately. IL-35 (20 ng/ml) was added to each replicate of these plates. In the control group, we only co-cultured 50 islets and 2 x 10^5^ CD4+ T cells without any treatment. All T cells were stimulated with plate-bound anti-CD28 (2 μg/ml) and anti-CD3 (5 μg/ml) as previously described [42]. IL-2 (500 U/ml) were added to each plate for cell growth and replenished every time the culture medium was changed (every other day). The culture medium used was RPMI 1640 (Sigma-Aldrich, St. Louis, MO, USA) supplemented with 10% fetal calf serum (Sigma-Aldrich), streptomycin (0.1 mg/ml; Sigma-Aldrich), L-glutamine and benzylpenicillin (100 U/ml, Roche Diagnostics Scandinavia, Bromma, Sweden). The culture medium was changed every second day. The function and survival state of islet cells were analyzed and the amount of Treg and Th17 in each group were quantified on days 1, 3 and 5 after co-culture. In the plates of the Treg group, we labeled the Th17 cells with CFSE before co-culture, and then detected the proliferation of these cells on day 5 after co-culture. Every experiment described above was repeated at least three times.

### Statistical analysis

Statistical analysis was performed using the GraphPad Software 6.0 (CA, USA). Comparisons between two groups were performed using unpaired t-tests. Mann-Whitney Rank Sum Tests were used for nonparametric observations. A p-value below 0.05 was considered statistically significant.

## Results

### IL-35 down-regulated the Th17/Treg ratio in the co-culture system

**Fig 1:**
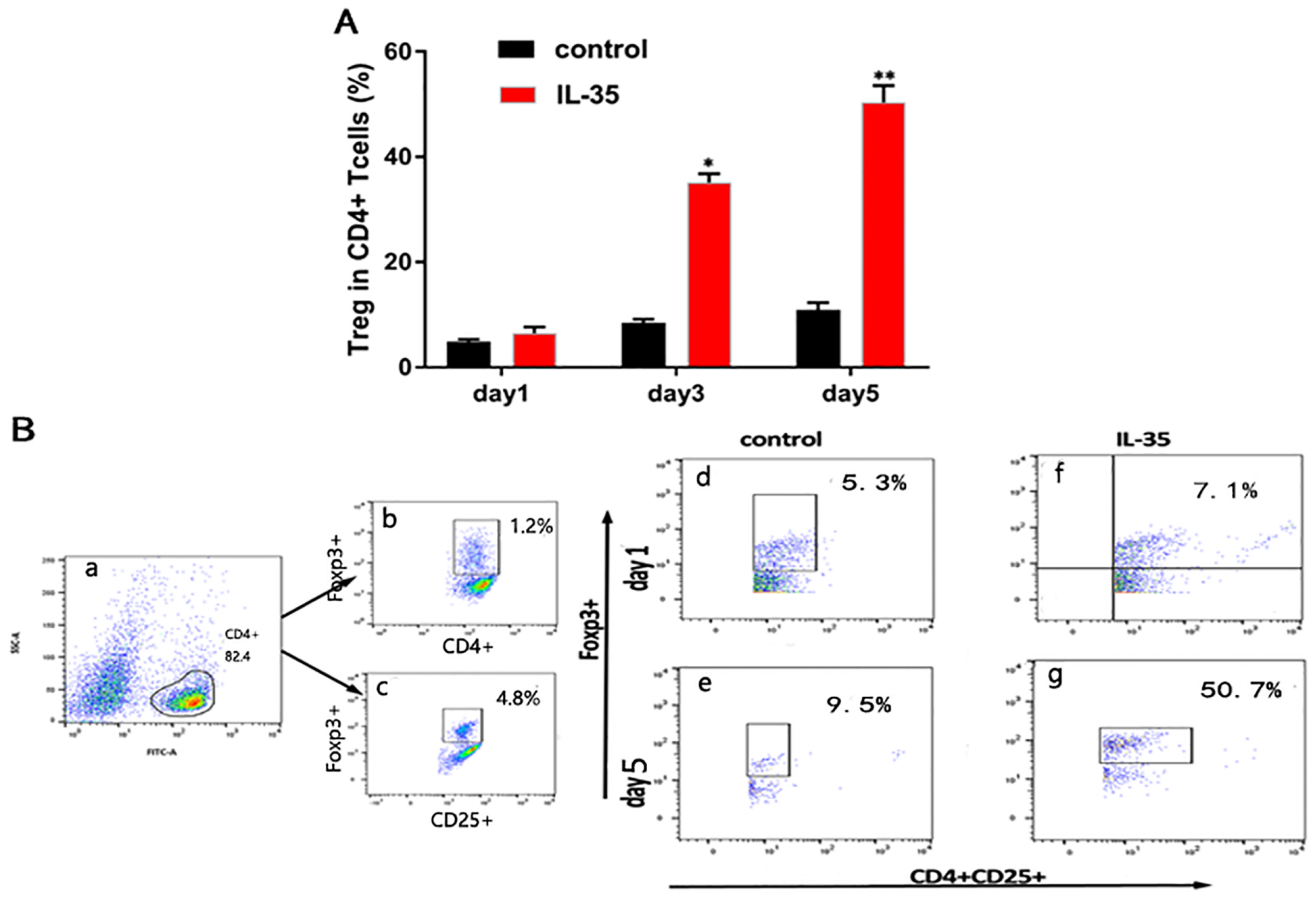
IL-35 increased the number of Treg in CD4+ T cells. (A)The ratio of Treg in CD4+ T cells. (B) Representative diagram showing in comparison with the control group, IL-35 markedly enhanced the proliferation of Tregs (50.7% in IL-35 group vs. 9.5% in control group on day 5, P<0.01) under these conditions with time. Results are expressed as means ± SEM, from two experiments (n = 3 times/group/experiment). Unpaired t-tests were performed for comparisons between control- and IL-35- groups on corresponding days. *, ** and *** denote p < 0.05, p < 0.01, and p < 0.001, respectively.

**Fig 2:**
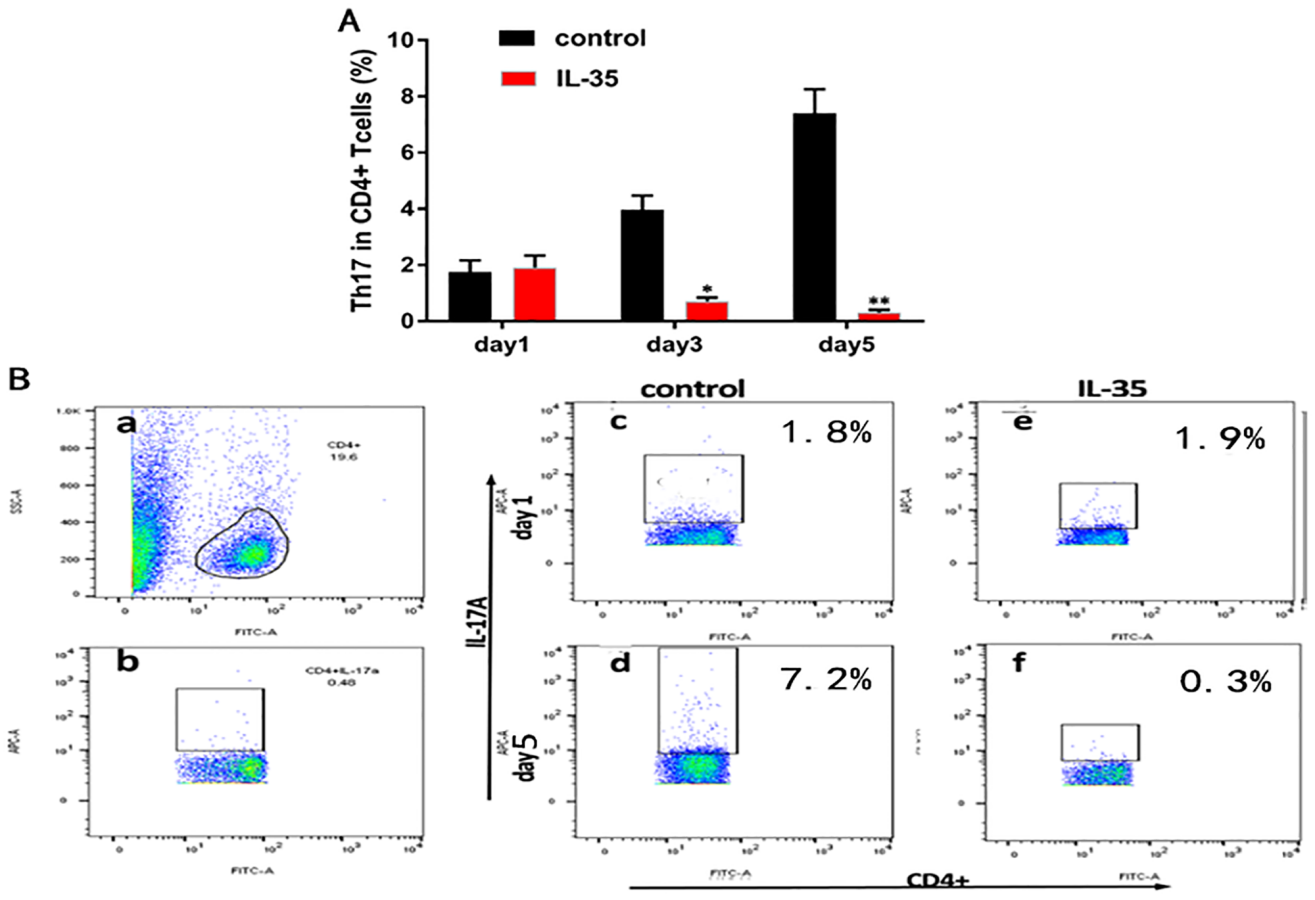
IL-35 decreased the prevalence of Th17 in CD4+ T cells. (A)The ratio of Th17 in CD4+ T cells. (B) Representative diagram showing Th17’s ratio in CD4+ T cells was significantly decreased in the IL-35 group compared to that in the control group (0.3% vs. 7.2% on day 5, P<0.01). Results are expressed as means ± SEM, from two experiments (n = 3 times/group/experiment). Unpaired t-tests were performed for comparisons between control- and IL-35- groups on corresponding days. *, ** and *** denote p < 0.05, p < 0.01, and p < 0.001, respectively.

**Fig 3:**
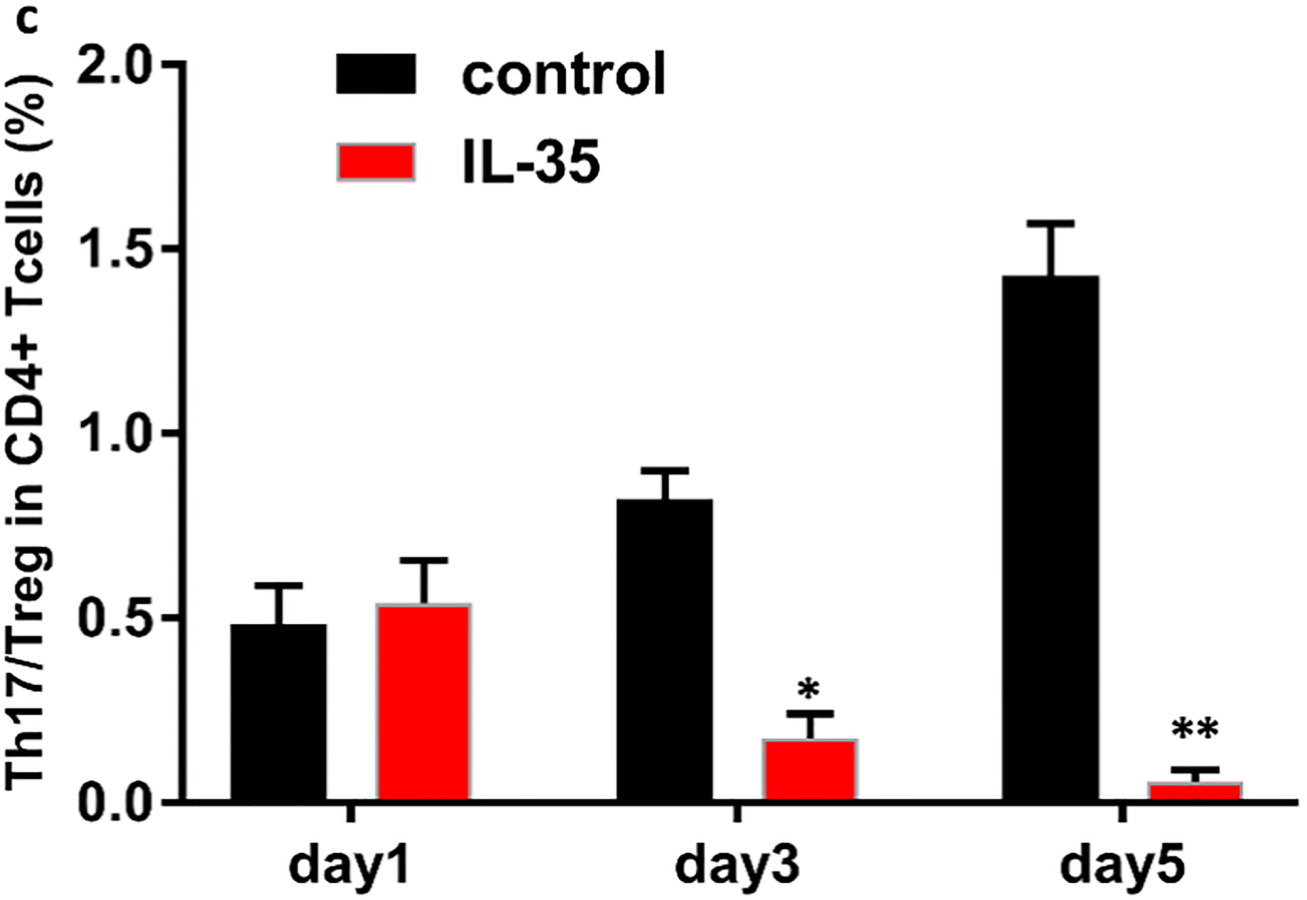
IL-35 down-regulated the ratio of Th17/Treg in CD4+ T cells. The ratio of Th17/Treg in CD4+ T cells in IL-35 group or control group on day 1, 3, 5 after co-culturing. A remarkable difference in the ratio of Th17/Treg in CD4+ T cells was observed under these conditions, particularly on day 5 after co-culture (1.4% in control group vs. 0.1% in IL-35 group, P<0.01). Results are expressed as means ± SEM, from two experiments (n = 3 times/group/experiment). Unpaired t-tests were performed for comparisons between control- and IL-35- groups on corresponding days. *, ** and *** denote p < 0.05, p < 0.01, and p < 0.001, respectively.

To determine the role of IL-35 in the CD4+ T cell subset, we purified CD4+CD25+, IL-17A+, and CD4+CD25- T cells from the spleen of C57BL/6 mice, and cultured these cells *in vitro* with plate-bound anti-CD3/CD28 antibodies and appropriate amount of IL-2. Results showed that in comparison with the control group, IL-35 markedly enhanced the proliferation of Tregs (50.7% in IL-35 group vs. 9.5% in control group on day 5, P<0.01) under these conditions with time (Fig 1). In contrast, although the absolute counts of Th17 showed only a slight increase, their ratio in CD4+ T cells was significantly decreased in the IL-35 group compared to that in the control group (0.3% vs. 7.2% on day 5, P<0.01) (Fig 2). Thus, a remarkable difference in the ratio of Th17/Treg in CD4+ T cells was observed under these conditions, particularly on day 5 after co-culture (1.4% in control group vs. 0.1% in IL-35 group, P<0.01) (Fig 3). Taken together, IL-35 down-regulated the Th17/Treg ratio in the co-culture system.

### IL-35 ameliorated the function of islet cells

**Fig 4:**
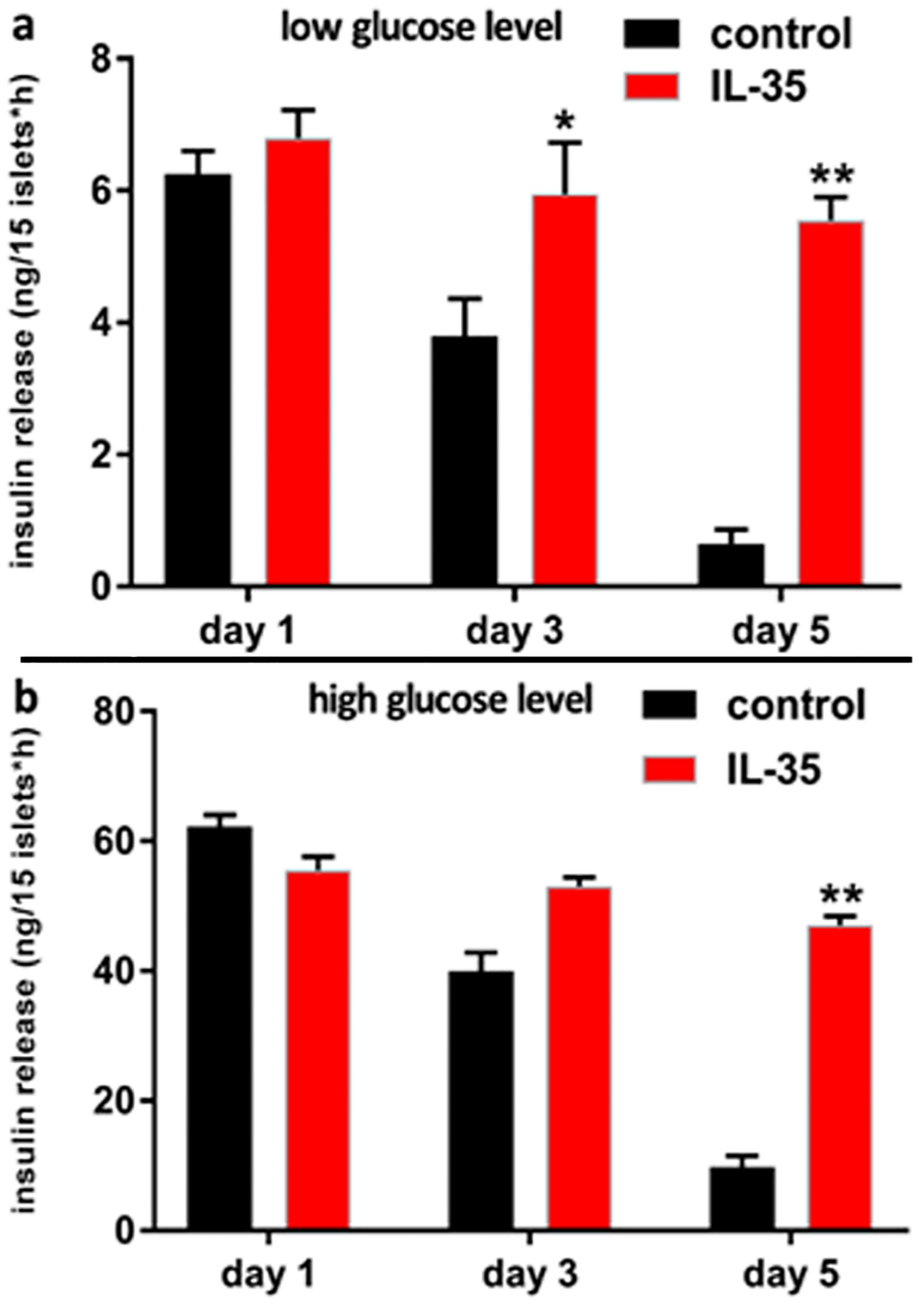
IL-35 ameliorated the function of islets in co-culture system. The amount of insulin release of 15 co-cultured islets in low glucose level (a) and in high glucose level (b). Results are expressed as means ± SEM, from two experiments (n = 3 times/group/experiment). Unpaired t-tests were performed for comparisons between control- and IL-35- groups on corresponding days. *, ** and *** denote p < 0.05, p < 0.01, and p < 0.001, respectively.

**Fig 5:**
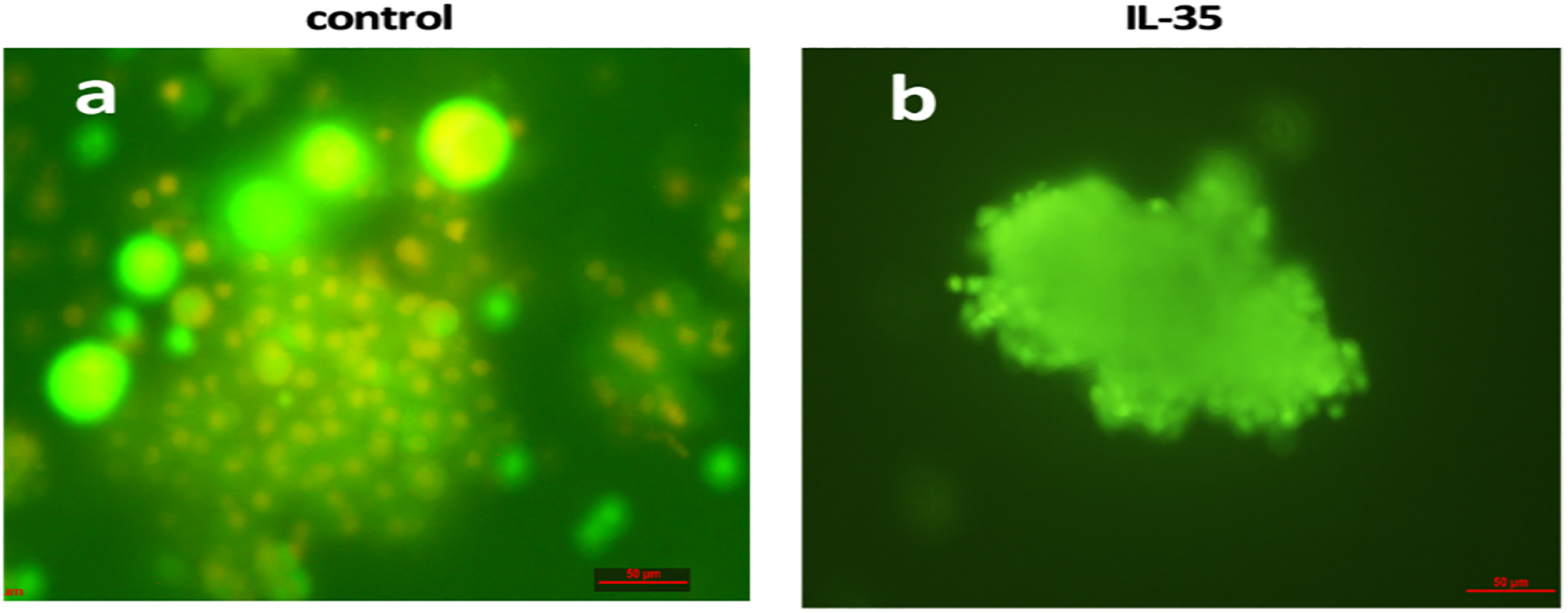
IL-35 delayed the survival of co-cultured islets. The islet cells were stained using AO/EB staining kit. The dead islet cells were labeled with yellow fluorescence, while the living islet cells were labeled with green fluorescence. In control group, most of islet cells were dead (a); however, the islet cells in IL-35 group were still living (b).

We next analyzed the function of islet cells under these conditions. Results showed that at a low glucose level (2.5 mM), insulin release of islet cells in the IL-35 group was markedly higher than that in the control group on day 5 (5.3 ng/15 islets*h in IL-35 group vs. 0.7 ng/15 islets*h in control group, P<0.01) (Fig 4a); similar results were observed at a high glucose level (16.7 mM) on day 5 (46.2 ng/15 islets*h in IL-35 group vs. 9.8 ng/15 islets*h in control group, P<0.01) (Fig 4b). Results of acridine orange (AO)/ethidium bromide (EB) staining also showed that IL-35 delayed the survival of co-cultured islet cells (Fig 5). Altogether, IL-35 treatment evidently ameliorated the insulin secretory function of islet cells.

### Increasing Treg ratio in CD4+ T cells ameliorated the function of islet cells

**Fig 6:**
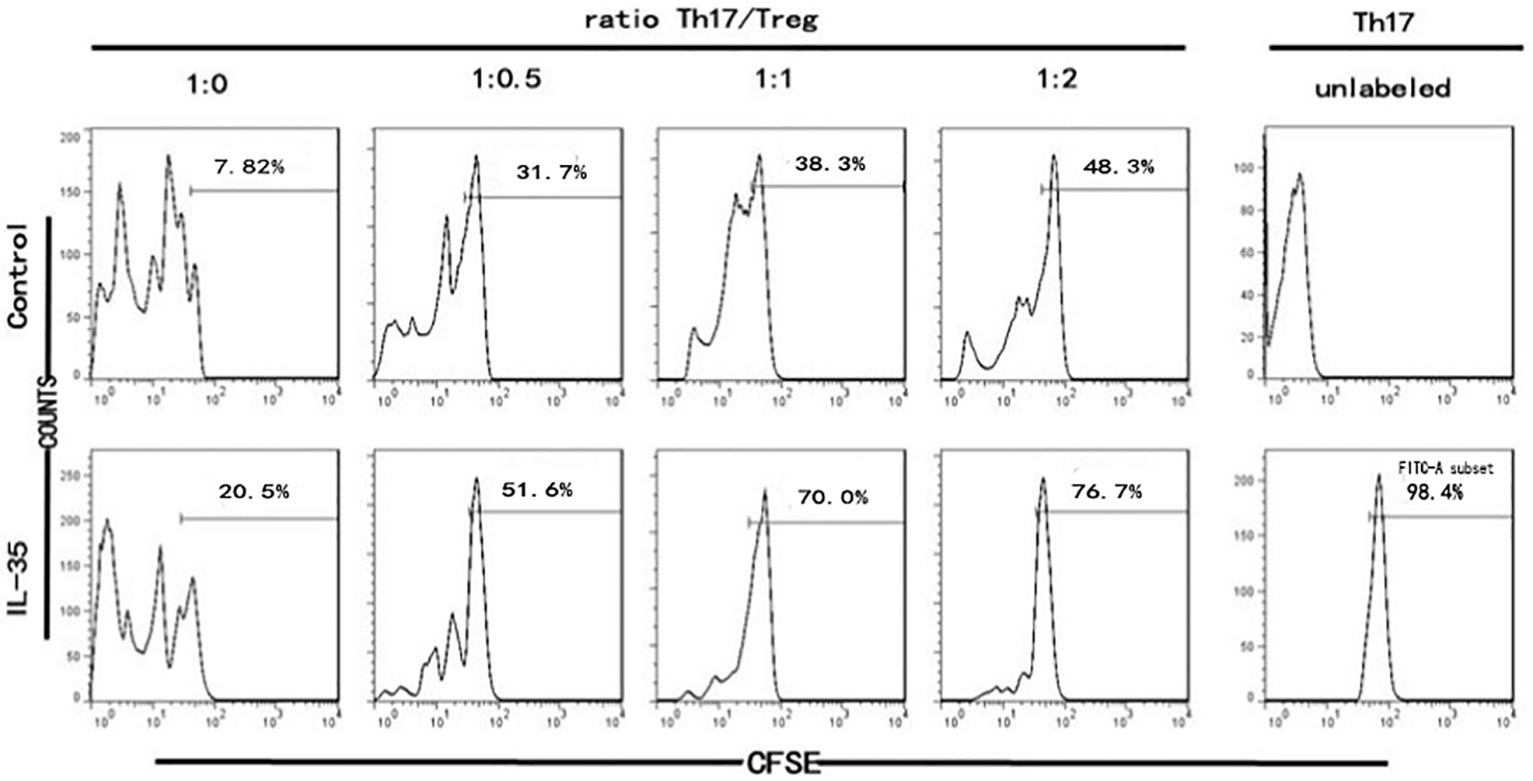
Treg inhibited the proliferation of Th17 and IL-35 enhanced the suppressive function of Treg. Representative histograms showing different Th17/Treg ratio (1:0, 1:0.5, 1:1, 1:2) in the co-culture system. With an increase of Treg ratio, on day 5, the proliferation of the CFSE-labeled Th17 cells was suppressed markedly (suppression ratio rising from 7.82% in 1:0 group to 48.2% in 1:2 group); besides, stronger suppression was observed when IL-35 was added, compared to that in the control group (suppression ratio rising from 20.5% in 1:0 group to 76.7% in 1:2 group).

**Fig 7:**
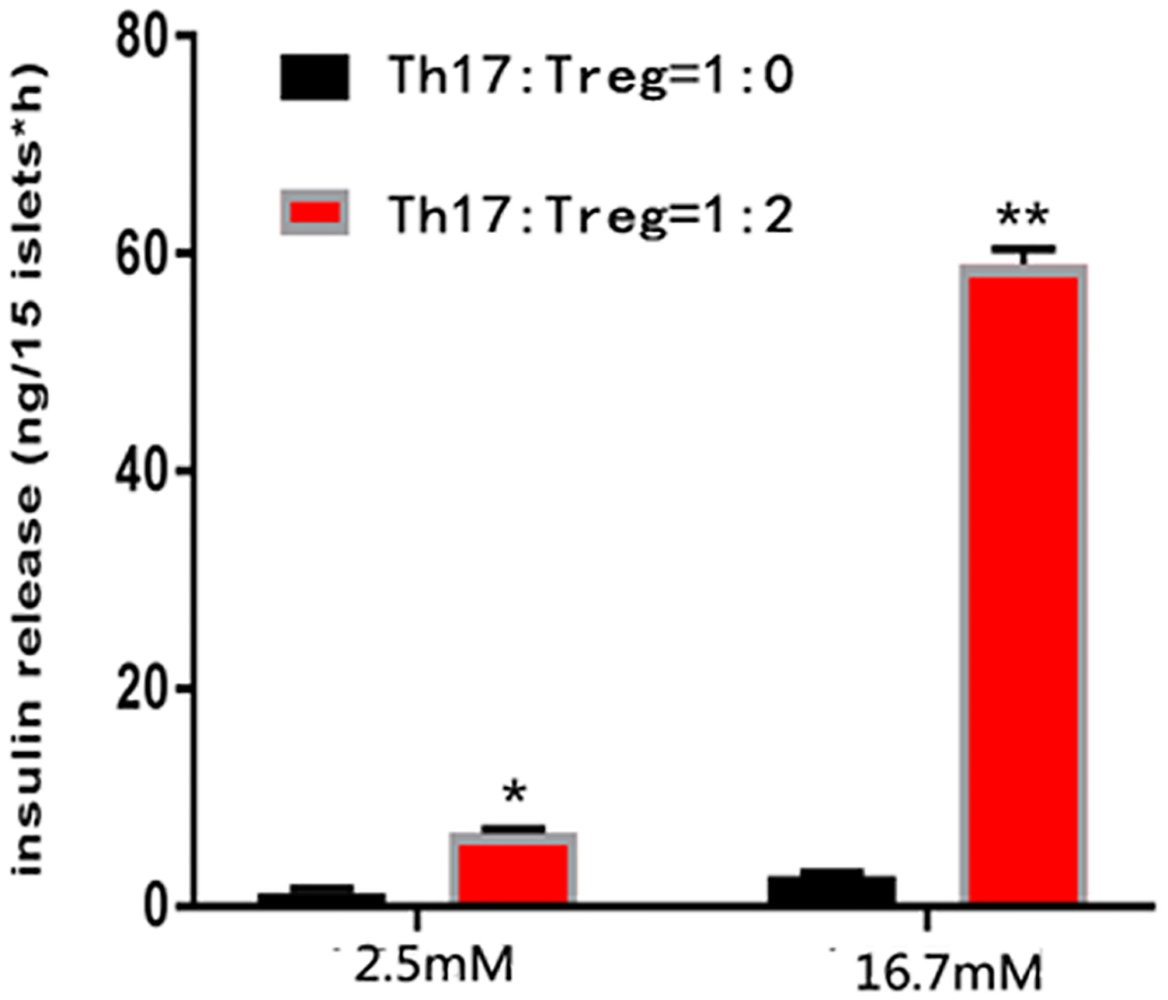
Up-regulating the prevalence of Treg ameliorated the function of islets. The function of co-cultured islet cells in Th17:Treg = 1:0 group and Th17:Treg = 1:2 group in low glucose level (2.5mM) or high glucose level (16.7mM). The results showed that regardless of the glucose level, islet cells in the Th17/Treg = 1:2 group had better insulin secretory function than that in the Th17/Treg = 1:0 group. Results are expressed as means ± SEM, from two experiments (n = 3 times/group/experiment). Unpaired t-tests were performed for comparisons between control- and IL-35- groups on corresponding days. *, ** and *** denote p < 0.05, p < 0.01, and p < 0.001, respectively.

Next, we decided to determine whether the improved function of islet cells was affected by the IL-35-mediated regulation of Th17/Treg ratio. For this, we modulated the Th17/Treg ratio (1:0, 1:0.5, 1:1, 1:2) in the co-culture system. We found that, on day 5, with an increase of Treg ratio, the proliferation of the CFSE-labeled Th17 cells was suppressed markedly (suppression ratio rising from 7.82% in 1:0 group to 48.2% in 1:2 group); besides, stronger suppression was observed when IL-35 was added, compared to that in the control group (Fig 6). Additionally, we analyzed the function of islet cells in Th17:Treg = 1:0 group and Th17:Treg = 1:2 group and found that regardless of the glucose level, islet cells in the Th17/Treg = 1:2 group had better insulin secretory function and survival rate than that in the Th17/Treg = 1:0 group (Fig 7).

## Discussion

Despite decades of investigation, mitigation of transplant immune rejection with less severe complications remains a challenge. Traditional clinical anti-rejection drugs, such as cyclosporin A and tacrolimus, comprehensively inhibit T cell activity by mainly binding to calcineurin of the cells and suppressing IL-2 release, which leads to numerous severe adverse effects [53]. Thus, it is important to find new drugs that can be specifically directed against specific T sub-populations on anti-transplanted rejection and result in less adverse effects [3].

Recently, CD4+ T cell sub-populations, Treg and Th17 cells, have drawn increased attention, and emerging evidence shows that the novel cytokine IL-35 and regulation of the ratio of these cell types play an important role in the development of autoimmunity and immune tolerance [11, 14, 45, 54–57]. Therefore, in the present study, we aimed to explore whether this mechanism exists in the development of mouse islet transplant rejection.

**Fig 8:**
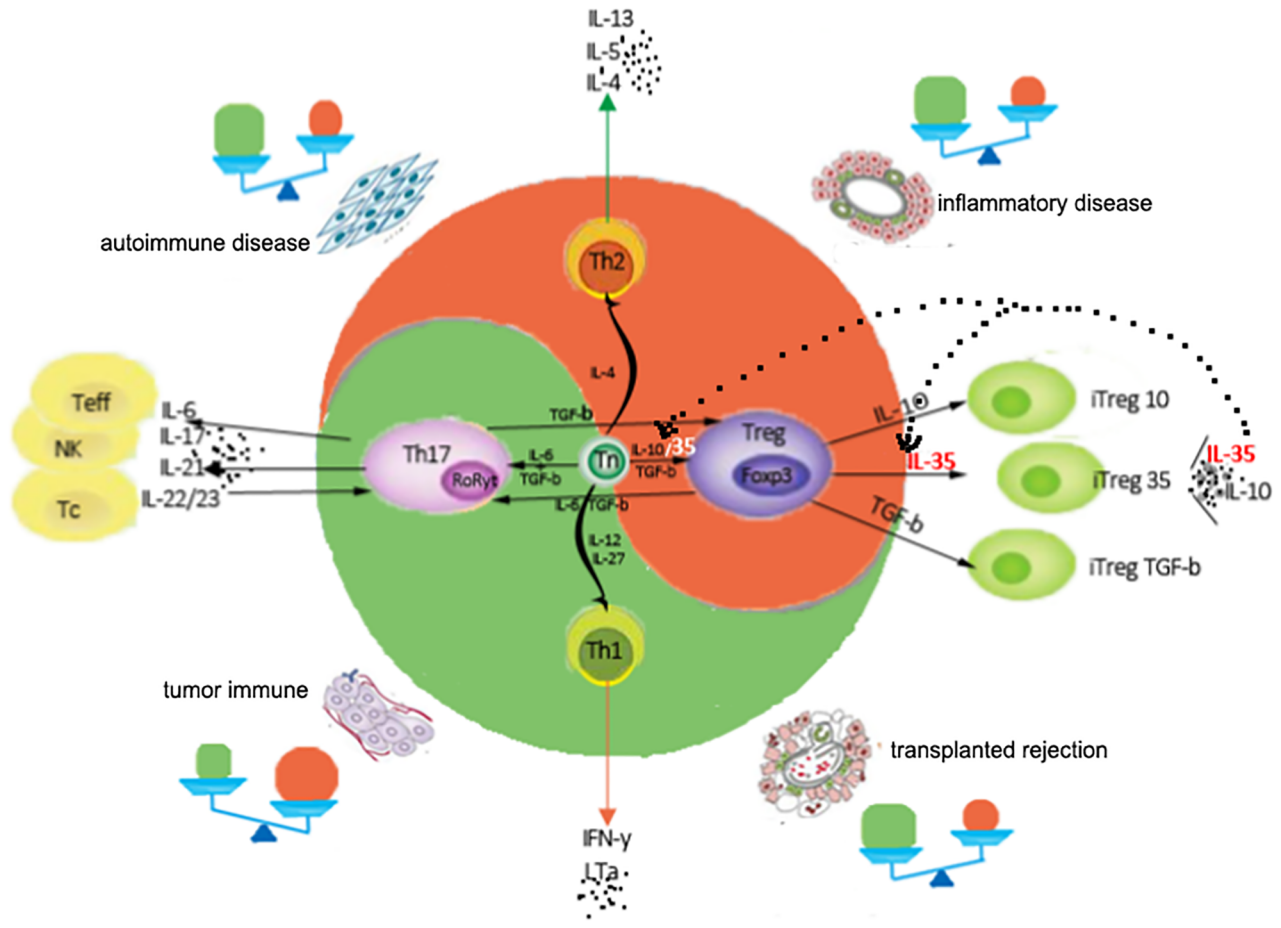
Model of IL-35 regulating Th17/Treg differentiation.

We found that the cell counts of Th17 and Treg cells were increased in the co-culture model and an increasing percentage of Tregs could inhibit the proliferation of Th17 cells. Similar results have been found in other studies, such as those for acute lung injury [31], *M*. *neoaurum* infection [33] and inflammation [58]. On one hand, the proliferation and growth of Tregs consume limiting T-cell growth factors such as IL-2 and release inhibitory cytokines such as IL-10, IL-35, and TGF-β on other immune cells, including Th17. On the other hand, differentiation of naïve CD4+ T cells towards each subset depends on the local cytokine environment. TGF-β is essential for the development of Treg and Th17 and IL-2 inhibits the polarization of Th17 cells. Co-availability of both TGF-β and IL-6 leads to the differentiation of naïve CD4+ T cells towards Th17; only TGF-β overdose (derived from added Tregs) favors the differentiation of Th17 into Tregs (Fig 8), thus decreasing the ratio of Th17 in CD4+ T cells. Furthermore, we found that a decrease in Th17/Treg ratio improved the function of transplanted islets, which was consistent with previous reports. Wu et al. [4] found that *ex vivo* expanded human Tregs in a humanized mouse model could improve the survival status of an islet allograft. A study from Canada showed that Treg cells could be recruited to transplanted islets, to suppress the activation of effector T-cells, and furthermore to induce alloantigen-specific tolerance [59]. Some results from an international co-operation group (named the ONE study) supported that Treg therapy can prevent immunological rejection of transplanted organs without the need for long-term use of pharmacological immunosuppression agents [60]. However, a study from Korea showed that in the peri-transplantation period, autologous Tregs infusion failed to induce transplanted immune tolerance in islet xenotransplantation settings (pig to non-human primates) [9]. The study suggested that there might be certain limitations on Tregs in inducing islet-transplanted tolerance and further study is needed in this context.

Moreover, we found that IL-35 *in vitro* treatment could down-regulate the ratio of Th17/Treg and prevent islet allograft failure. The same trend could be found in stem cell transplantation [61], T1D [41, 43] and acute myeloid leukemia blasts [62]. However, the role of IL-35 in solid organ transplantation is poorly understood. Studies have demonstrated the role of IL-35 in conversion of human and murine CD4+CD25- T cells into IL-35-induced Treg (iTr35) cells [63–65]. Ma et al. showed that rhIL-35 could induce the expression of EBI3 and P35 in CD4+CD25- T cells (including Th17 cells) and relative Tregs were capable of inducing a further increase in IL-35 levels [63]. IL-35 treatment activated the phosphorylation of STAT1 and STAT3 in CD4+ T cells, which then allowed the differentiation of naïve CD4+ T cells into Tregs. Additionally, exogenous IL-35 also favored the conversion of Tregs into iTr35 cells. Interestingly, the induced iTr35 cells still possessed the ability to release IL-35 and induce a positive feedback to promote CD4+CD25- T cell differentiation into Tregs and IL-35 production [37–39, 42, 45, 66]. All these processes are involved in islet transplant rejection (Fig 7). Furthermore, as demonstrated by Kailash et al. [41], IL-35 administration could counteract established T1D. Although the exact mechanism is still unknown, several mechanisms might be involved. According to Kailash et al., Tregs might play a role, thus preventing the autoimmune destruction of β cells of patients. Moreover, it is suggested that IL-35 might promote the differentiation of other pancreatic cells (e.g. α cells) into β-cells through the GABA pathway [67, 68] or other signaling pathways *in vivo*. Taken together, IL-35 might not only improve islet transplanted rejection, but also can help reverse the destruction of β cells of T1D patients themselves.

## Conclusions

IL-35 mitigates the function of murine transplanted islet cells via regulation of the Th17/Treg ratio. This might serve as a potential and promising therapeutic strategy for islet transplant rejection and T1D, thus raising the need for conducting more in-vivo studies in this context.

## Supporting information

S1 Table NC3Rs ARRIVE guidelines checklist (fillable). (PDF)

## Abbreviations

CBD: common bile duct
CFSE: carboxyfluorescein diacetate succinimidyl ester
Ebi3: Epstein-Barr-virus-induced gene 3
ELISA: enzyme-linked immunosorbent assay
FoxP3: Forkhead box P3
GSIS: glucose-stimulated insulin secretion
IL-35: Interleukin-35
PIT: pancreatic islet transplantation
RORγt: retinoic acid receptor-related orphan receptor γt
STAT5: signal transducer and activator of transcription 5
T1D: type 1 diabetes
Th1: T helper 1 cell
Th17: T helper 17 cell
Th2: T helper 2 cell
Treg: regulatory CD4+CD25+Foxp3+ T cell

## References

[1] Ahearn AJ, Parekh JR, Posselt AM. Islet transplantation for Type 1 diabetes: where are we now? Expert Rev Clin Immunol. 2015;11(1):59–68. doi: 10.1586/1744666X.2015.978291 . 25454816 10.1586/1744666X.2015.978291

[2] Ben Nasr M, D'Addio F, Usuelli V, Tezza S, Abdi R, Fiorina P. The rise, fall, and resurgence of immunotherapy in type 1 diabetes. Pharmacol Res. 2015;98:31–8. doi: 10.1016/j.phrs.2014.07.004 . 25107501 10.1016/j.phrs.2014.07.004

[3] van der Net JB, Bushell A, Wood KJ, Harden PN. Regulatory T cells: first steps of clinical application in solid organ transplantation. Transpl Int. 2016;29(1):3–11. doi: 10.1111/tri.12608 . 25981203 10.1111/tri.12608

[4] Wu DC, Hester J, Nadig SN, Zhang W, Trzonkowski P, Gray D, et al Ex vivo expanded human regulatory T cells can prolong survival of a human islet allograft in a humanized mouse model. Transplantation. 2013;96(8):707–16. doi: 10.1097/TP.0b013e31829fa271 23917725 10.1097/TP.0b013e31829fa271 PMC3864182

[5] Petrelli A, Carvello M, Vergani A, Lee KM, Tezza S, Du M, et al IL-21 is an antitolerogenic cytokine of the late-phase alloimmune response. Diabetes. 2011;60(12):3223–34. doi: 10.2337/db11-0880 22013017 10.2337/db11-0880 PMC3219943

[6] Vergani A, D'Addio F, Jurewicz M, Petrelli A, Watanabe T, Liu K, et al A novel clinically relevant strategy to abrogate autoimmunity and regulate alloimmunity in NOD mice. Diabetes. 2010;59(9):2253–64. doi: 10.2337/db09-1264 20805386 10.2337/db09-1264 PMC2927948

[7] Tezza S, Ben Nasr M, Vergani A, Valderrama Vasquez A, Maestroni A, Abdi R, et al Novel immunological strategies for islet transplantation. Pharmacological Research. 2015;98:69–75. doi: 10.1016/j.phrs.2014.06.016 25014184 10.1016/j.phrs.2014.06.016

[8] Hering BJ, Bellin MD. Transplantation: Sustained benefits of islet transplants for T1DM. Nat Rev Endocrinol. 2015;11(10):572–4. doi: 10.1038/nrendo.2015.126 . 26239608 10.1038/nrendo.2015.126

[9] Shin JS, Min BH, Kim JM, Kim JS, Yoon IH, Kim HJ, et al Failure of transplantation tolerance induction by autologous regulatory T cells in the pig-to-non-human primate islet xenotransplantation model. Xenotransplantation. 2016;23(4):300–9. doi: 10.1111/xen.12246 . 27387829 10.1111/xen.12246

[10] Singh K, Hjort M, Thorvaldson L, Sandler S. Concomitant analysis of Helios and Neuropilin-1 as a marker to detect thymic derived regulatory T cells in naive mice. Sci Rep. 2015;5:7767 doi: 10.1038/srep07767 25586548 10.1038/srep07767 PMC4293597

[11] Stangou M, Bantis C, Skoularopoulou M, Korelidou L, Kouloukouriotou D, Scina M, et al Th1, Th2 and Treg/T17 cytokines in two types of proliferative glomerulonephritis. Indian J Nephrol. 2016;26(3):159–66. doi: 10.4103/0971-4065.159303 27194829 10.4103/0971-4065.159303 PMC4862260

[12] Sakaguchi S, Yamaguchi T, Nomura T, Ono M. Regulatory T cells and immune tolerance. Cell. 2008;133(5):775–87. doi: 10.1016/j.cell.2008.05.009 . 18510923 10.1016/j.cell.2008.05.009

[13] Littman DR, Rudensky AY. Th17 and regulatory T cells in mediating and restraining inflammation. Cell. 2010;140(6):845–58. doi: 10.1016/j.cell.2010.02.021 . 20303875 10.1016/j.cell.2010.02.021

[14] Nguyen MT, Fryml E, Sahakian SK, Liu S, Cantarovich M, Lipman M, et al Pretransplant Recipient Circulating CD4+CD127lo/- Tumor Necrosis Factor Receptor 2+ Regulatory T Cells: A Surrogate of Regulatory T Cell-Suppressive Function and Predictor of Delayed and Slow Graft Function After Kidney Transplantation. Transplantation. 2016;100(2):314–24. doi: 10.1097/TP.0000000000000942 . 26425877 10.1097/TP.0000000000000942

[15] Guinan EC, Cole GA, Wylie WH, Kelner RH, Janec KJ, Yuan H, et al Ex Vivo Costimulatory Blockade to Generate Regulatory T Cells From Patients Awaiting Kidney Transplantation. Am J Transplant. 2016;16(7):2187–95. doi: 10.1111/ajt.13725 . 26790369 10.1111/ajt.13725

[16] Xu X, Huang H, Wang Q, Cai M, Qian Y, Han Y, et al IFN-gamma-producing Th1-like regulatory T cells may limit acute cellular renal allograft rejection: Paradoxical post-transplantation effects of IFN-gamma. Immunobiology. 2017;222(2):280–90. doi: 10.1016/j.imbio.2016.09.012 . 27665996 10.1016/j.imbio.2016.09.012

[17] Hu M, Wang YM, Wang Y, Zhang GY, Zheng G, Yi S, et al Regulatory T cells in kidney disease and transplantation. Kidney Int. 2016;90(3):502–14. doi: 10.1016/j.kint.2016.03.022 . 27263492 10.1016/j.kint.2016.03.022

[18] Todo S, Yamashita K, Goto R, Zaitsu M, Nagatsu A, Oura T, et al A pilot study of operational tolerance with a regulatory T-cell-based cell therapy in living donor liver transplantation. Hepatology. 2016;64(2):632–43. doi: 10.1002/hep.28459 . 26773713 10.1002/hep.28459

[19] Boix-Giner F, Millan O, San Segundo D, Munoz-Cacho P, Mancebo E, Llorente S, et al High frequency of central memory regulatory T cells allows detection of liver recipients at risk of early acute rejection within the first month after transplantation. Int Immunol. 2016;28(2):55–64. doi: 10.1093/intimm/dxv048 26270267 10.1093/intimm/dxv048 PMC4885217

[20] Taubert R, Danger R, Londono MC, Christakoudi S, Martinez-Picola M, Rimola A, et al Hepatic Infiltrates in Operational Tolerant Patients After Liver Transplantation Show Enrichment of Regulatory T Cells Before Proinflammatory Genes Are Downregulated. Am J Transplant. 2016;16(4):1285–93. doi: 10.1111/ajt.13617 . 26603835 10.1111/ajt.13617

[21] Safinia N, Vaikunthanathan T, Fraser H, Thirkell S, Lowe K, Blackmore L, et al Successful expansion of functional and stable regulatory T cells for immunotherapy in liver transplantation. Oncotarget. 2016;7(7):7563–77. doi: 10.18632/oncotarget.6927 26788992 10.18632/oncotarget.6927 PMC4884938

[22] Haarer J, Riquelme P, Hoffmann P, Schnitzbauer A, Schlitt HJ, Sawitzki B, et al Early Enrichment and Restitution of the Peripheral Blood Treg Pool Is Associated With Rejection-Free Stable Immunosuppression After Liver Transplantation. Transplantation. 2016;100(7):e39–40. doi: 10.1097/TP.0000000000001190 . 27326814 10.1097/TP.0000000000001190

[23] Ezzelarab MB, Zhang H, Guo H, Lu L, Zahorchak AF, Wiseman RW, et al Regulatory T Cell Infusion Can Enhance Memory T Cell and Alloantibody Responses in Lymphodepleted Nonhuman Primate Heart Allograft Recipients. Am J Transplant. 2016;16(7):1999–2015. doi: 10.1111/ajt.13685 26700196 10.1111/ajt.13685 PMC4919255

[24] Mirza K, Gustafsson F, Gullestad L, Arora S, Andersen C. Effect of everolimus initiation and early calcineurin inhibitor withdrawal on myocardial FOXP3+ regulatory T cells in heart transplantation. Transpl Immunol. 2016;38:75–7. doi: 10.1016/j.trim.2016.05.004 . 27260644 10.1016/j.trim.2016.05.004

[25] Yang XO, Pappu BP, Nurieva R, Akimzhanov A, Kang HS, Chung Y, et al T helper 17 lineage differentiation is programmed by orphan nuclear receptors ROR alpha and ROR gamma. Immunity. 2008;28(1):29–39. doi: 10.1016/j.immuni.2007.11.016 18164222 10.1016/j.immuni.2007.11.016 PMC2587175

[26] Bettelli E, Oukka M, Kuchroo VK. T(H)-17 cells in the circle of immunity and autoimmunity. Nat Immunol. 2007;8(4):345–50. doi: 10.1038/ni0407-345 . 17375096 10.1038/ni0407-345

[27] Ivanov II, McKenzie BS, Zhou L, Tadokoro CE, Lepelley A, Lafaille JJ, et al The orphan nuclear receptor RORgammat directs the differentiation program of proinflammatory IL-17+ T helper cells. Cell. 2006;126(6):1121–33. doi: 10.1016/j.cell.2006.07.035 . 16990136 10.1016/j.cell.2006.07.035

[28] Dong C. Diversification of T-helper-cell lineages: finding the family root of IL-17-producing cells. Nat Rev Immunol. 2006;6(4):329–33. doi: 10.1038/nri1807 . 16557264 10.1038/nri1807

[29] Bettelli E, Korn T, Kuchroo VK. Th17: the third member of the effector T cell trilogy. Curr Opin Immunol. 2007;19(6):652–7. doi: 10.1016/j.coi.2007.07.020 17766098 10.1016/j.coi.2007.07.020 PMC2288775

[30] Zhang J, Lin Y, Li C, Zhang X, Cheng L, Dai L, et al IL-35 Decelerates the Inflammatory Process by Regulating Inflammatory Cytokine Secretion and M1/M2 Macrophage Ratio in Psoriasis. J Immunol. 2016 doi: 10.4049/jimmunol.1600446 . 27527600 10.4049/jimmunol.1600446

[31] Zhang F, Li MY, Lan YT, Wang CB. Imbalance of Th17/Tregs in rats with smoke inhalation-induced acute lung injury. Sci Rep. 2016;6:21348 doi: 10.1038/srep21348 . 26884314 10.1038/srep21348 PMC4756332

[32] Brucklacher-Waldert V, Steinbach K, Lioznov M, Kolster M, Holscher C, Tolosa E. Phenotypical characterization of human Th17 cells unambiguously identified by surface IL-17A expression. J Immunol. 2009;183(9):5494–501. doi: 10.4049/jimmunol.0901000 . 19843935 10.4049/jimmunol.0901000

[33] Wang CF, Yang WT, Yue LM, Qiu JY, Zhang LJ, Wang C, et al Prominent contribution of Th1, Th17, and Tregs to the host response during M. neoaurum infection. Genet Mol Res. 2016;15(3). doi: 10.4238/gmr.15038989 . 27706786 10.4238/gmr.15038989

[34] Salman J, Ius F, Knoefel AK, Sommer W, Siemeni T, Kuehn C, et al Association of higher CD4+ CD25high CD127low, FoxP3+, and IL-2+ T cell frequencies early after lung transplantation with less chronic lung allograft dysfunction at two years. Am J Transplant. 2016 doi: 10.1111/ajt.14148 . 27931084 10.1111/ajt.14148

[35] Kappen JH, Durham SR, Veen HI, Shamji MH. Applications and mechanisms of immunotherapy in allergic rhinitis and asthma. Ther Adv Respir Dis. 2016 doi: 10.1177/1753465816669662 . 27678500 10.1177/1753465816669662 PMC5941975

[36] Haribhai D, Chatila TA, Williams CB. Immunotherapy with iTreg and nTreg Cells in a Murine Model of Inflammatory Bowel Disease. Methods Mol Biol. 2016;1422:197–211. doi: 10.1007/978-1-4939-3603-8_19 . 27246035 10.1007/978-1-4939-3603-8_19

[37] Collison LW, Workman CJ, Kuo TT, Boyd K, Wang Y, Vignali KM, et al The inhibitory cytokine IL-35 contributes to regulatory T-cell function. Nature. 2007;450(7169):566–9. doi: 10.1038/nature06306 . 18033300 10.1038/nature06306

[38] Collison LW, Chaturvedi V, Henderson AL, Giacomin PR, Guy C, Bankoti J, et al IL-35-mediated induction of a potent regulatory T cell population. Nat Immunol. 2010;11(12):1093–101. doi: 10.1038/ni.1952 20953201 10.1038/ni.1952 PMC3008395

[39] Collison LW, Delgoffe GM, Guy CS, Vignali KM, Chaturvedi V, Fairweather D, et al The composition and signaling of the IL-35 receptor are unconventional. Nat Immunol. 2012;13(3):290–9. doi: 10.1038/ni.2227 22306691 10.1038/ni.2227 PMC3529151

[40] Collison LW, Pillai MR, Chaturvedi V, Vignali DA. Regulatory T cell suppression is potentiated by target T cells in a cell contact, IL-35- and IL-10-dependent manner. J Immunol. 2009;182(10):6121–8. doi: 10.4049/jimmunol.0803646 19414764 10.4049/jimmunol.0803646 PMC2698997

[41] Singh K. K E, Lindroos J., Hjort M., Lundberg M., Espes D., Carlsson P. O., Sandler S., Thorvaldson L. Interleukin-35 administration counteracts established murine type 1 diabetes—possible involvement of regulatory T cells. Sci Rep. 2015;5:12633 doi: 10.1038/srep12633 26224624 10.1038/srep12633 PMC4519737

[42] Niedbala W, Wei XQ, Cai B, Hueber AJ, Leung BP, McInnes IB, et al IL-35 is a novel cytokine with therapeutic effects against collagen-induced arthritis through the expansion of regulatory T cells and suppression of Th17 cells. Eur J Immunol. 2007;37(11):3021–9. doi: 10.1002/eji.200737810 . 17874423 10.1002/eji.200737810

[43] Manzoor F, Johnson MC, Li C, Samulski RJ, Wang B, Tisch R. beta-cell-specific IL-35 therapy suppresses ongoing autoimmune diabetes in NOD mice. Eur J Immunol. 2016 doi: 10.1002/eji.201646493 . 27859048 10.1002/eji.201646493 PMC5233468

[44] Kanai K, Park AM, Yoshida H, Tsunoda I, Yoshie O. IL-35 Suppresses Lipopolysaccharide-Induced Airway Eosinophilia in EBI3-Deficient Mice. J Immunol. 2016 doi: 10.4049/jimmunol.1600506 . 27881708 10.4049/jimmunol.1600506

[45] Guan SY, Leng RX, Khan MI, Qureshi H, Li XP, Ye DQ, et al Interleukin-35: a Potential Therapeutic Agent for Autoimmune Diseases. Inflammation. 2016 doi: 10.1007/s10753-016-0453-9 . 27696334 10.1007/s10753-016-0453-9

[46] Hu D. Role of Anti-inflammatory Cytokines IL-35 and IL-37 in Asthma. Inflammation. 2016 doi: 10.1007/s10753-016-0480-6 . 27878686 10.1007/s10753-016-0480-6

[47] Liu Y, Wu Y, Wang Y, Cai Y, Hu B, Bao G, et al IL-35 mitigates murine acute graft-versus-host disease with retention of graft-versus-leukemia effects. Leukemia. 2015;29(4):939–46. doi: 10.1038/leu.2014.310 25363669 10.1038/leu.2014.310 PMC4391991

[48] Rydgren T, Bengtsson D, Sandler S. Complete protection against interleukin-1beta-induced functional suppression and cytokine-mediated cytotoxicity in rat pancreatic islets in vitro using an interleukin-1 cytokine trap. Diabetes. 2006;55(5):1407–12. . 16644698 10.2337/db05-1273

[49] Song MM, Fang S, Tanaka S, Sugiyama K, Kiyomi A, Kato R, et al Effects of arsenic disulfide on proliferation, cytokine production, and frequencies of CD4(+), CD8(+), and regulatory T cells in mitogen-activated human peripheral blood mononuclear cells. Int Immunopharmacol. 2015;29(2):832–8. doi: 10.1016/j.intimp.2015.08.034 . 26359544 10.1016/j.intimp.2015.08.034

[50] Venken K, Thewissen M, Hellings N, Somers V, Hensen K, Rummens JL, et al A CFSE based assay for measuring CD4+CD25+ regulatory T cell mediated suppression of auto-antigen specific and polyclonal T cell responses. J Immunol Methods. 2007;322(1–2):1–11. doi: 10.1016/j.jim.2007.01.025 . 17368474 10.1016/j.jim.2007.01.025

[51] Mueller KR, Balamurugan AN, Cline GW, Pongratz RL, Hooper RL, Weegman BP, et al Differences in glucose-stimulated insulin secretion in vitro of islets from human, nonhuman primate, and porcine origin. Xenotransplantation. 2013;20(2):75–81. doi: 10.1111/xen.12022 23384163 10.1111/xen.12022 PMC4145818

[52] Baskin DG. A Historical Perspective on the Identification of Cell Types in Pancreatic Islets of Langerhans by Staining and Histochemical Techniques. J Histochem Cytochem. 2015;63(8):543–58. doi: 10.1369/0022155415589119 26216133 10.1369/0022155415589119 PMC4530402

[53] Zongyi Y, Baifeng L, Funian Z, Hao L, Xin W. Risk factors of acute kidney injury after orthotopic liver transplantation in China. Sci Rep. 2017;7:41555 doi: 10.1038/srep41555 28134286 10.1038/srep41555 PMC5278509

[54] Cebula A, Seweryn M, Rempala GA, Pabla SS, McIndoe RA, Denning TL, et al Thymus-derived regulatory T cells contribute to tolerance to commensal microbiota. Nature. 2013;497(7448):258–62. doi: 10.1038/nature12079 23624374 10.1038/nature12079 PMC3711137

[55] Zhang XH, Zhou Y, Xu LP, Han W, Chen H, Chen YH, et al Reduced IL-35 levels are associated with increased platelet aggregation and activation in patients with acute graft-versus-host disease after allogeneic hematopoietic stem cell transplantation. Annals of Hematology. 2015;94(5):837–45. doi: 10.1007/s00277-014-2278-7 25512184 10.1007/s00277-014-2278-7

[56] Roccatello D, Sciascia S, Di Simone D, Solfietti L, Naretto C, Fenoglio R, et al New insights into immune mechanisms underlying response to RTX in patients with membranous nephropathy: A prospective study and a review of the literature. Autoimmun Rev. 2016 doi: 10.1016/j.autrev.2016.02.014 . 26876383 10.1016/j.autrev.2016.02.014

[57] Zongyi Y, Dongying C, Baifeng L. Global Regulatory T-Cell Research from 2000 to 2015: A Bibliometric Analysis. PLoS One. 2016;11(9):e0162099 doi: 10.1371/journal.pone.0162099 27611317 10.1371/journal.pone.0162099 PMC5017768

[58] Luo T, Ji WJ, Yuan F, Guo ZZ, Li YX, Dong Y, et al Th17/Treg Imbalance Induced by Dietary Salt Variation Indicates Inflammation of Target Organs in Humans. Sci Rep. 2016;6:26767 doi: 10.1038/srep26767 27353721 10.1038/srep26767 PMC4926124

[59] Montane J. O M, Alvarez S., Bischoff L., Dai D. L., Soukhatcheva G., Priatel J. J., Hardenberg G., Levings M. K., Tan R., Orban P. C., Verchere C. B. CCL22 Prevents Rejection of Mouse Islet Allografts and Induces Donor-Specific Tolerance. Cell Transplant. 2015;24(10):2143–54. doi: 10.3727/096368914X685249 . 26423995 10.3727/096368914X685249

[60] A Unified Approach to Evaluating Cellular Immunotherapy in Solid Organ Transplantation 2017 [cited 2017 April 20]. http://www.onestudy.org/.

[61] Zhang X, Zhou Y, Xu L, Han W, Chen H, Chen Y, et al Reduced IL-35 levels are associated with increased platelet aggregation and activation in patients with acute graft-versus-host disease after allogeneic hematopoietic stem cell transplantation. Ann Hematol. 2015;94(5):837–45. doi: 10.1007/s00277-014-2278-7 . 25512184 10.1007/s00277-014-2278-7

[62] Tao Q, Pan Y, Wang Y, Wang H, Xiong S, Li Q, et al Regulatory T cells-derived IL-35 promotes the growth of adult acute myeloid leukemia blasts. Int J Cancer. 2015 doi: 10.1002/ijc.29563 . 25866142 10.1002/ijc.29563

[63] Ma Y, Chen L, Xie G, Zhou Y, Yue C, Yuan X, et al Elevated level of Interleukin-35 in colorectal cancer induces conversion of T cells into iTr35 by activating STAT1/STAT3. Oncotarget. 2016 doi: 10.18632/oncotarget.12193 . 27682874 10.18632/oncotarget.12193 PMC5341959

[64] Seyerl M, Kirchberger S, Majdic O, Seipelt J, Jindra C, Schrauf C, et al Human rhinoviruses induce IL-35-producing Treg via induction of B7-H1 (CD274) and sialoadhesin (CD169) on DC. Eur J Immunol. 2010;40(2):321–9. doi: 10.1002/eji.200939527 . 19950173 10.1002/eji.200939527

[65] Hanahan D, Weinberg RA. Hallmarks of cancer: the next generation. Cell. 2011;144(5):646–74. doi: 10.1016/j.cell.2011.02.013 . 21376230 10.1016/j.cell.2011.02.013

[66] Ye S, Wu J, Zhou L, Lv Z, Xie H, Zheng S. Interleukin-35: the future of hyperimmune-related diseases? J Interferon Cytokine Res. 2013;33(6):285–91. doi: 10.1089/jir.2012.0086 . 23472662 10.1089/jir.2012.0086

[67] Li J, Casteels T, Frogne T, Ingvorsen C, Honore C, Courtney M, et al Artemisinins Target GABAA Receptor Signaling and Impair alpha Cell Identity. Cell. 2016 doi: 10.1016/j.cell.2016.11.010 . 27916275 10.1016/j.cell.2016.11.010 PMC5236063

[68] Ben-Othman N, Vieira A, Courtney M, Record F, Gjernes E, Avolio F, et al Long-Term GABA Administration Induces Alpha Cell-Mediated Beta-like Cell Neogenesis. Cell. 2016 doi: 10.1016/j.cell.2016.11.002 . 27916274 10.1016/j.cell.2016.11.002

